# Auto-toxemia*Read at the annual meeting of the North Central Ohio Medical Society held in Mansfield Ohio, March 25, 1904.

**Published:** 1904-06

**Authors:** Robt. C. M. Lewis

**Affiliations:** Marion, Ohio, Member American Medical Association, Etc.


For Texas Medical Journal.

### Auto-toxemia.*

BY ROBT. C. M. LEWIS, M. D., MARION, OHIO,
Member American Medical Association, Etc.

In this paper I may not present anything new, for my subject—auto-toxemia—is old, though it may have flourished under other names during the making and unmaking of medical history.

Though old, it is a factor that must not be lost sight of when the question of diagnosis is to be considered. The conception of auto-toxemia is not merely an attractive hypothesis, since positive facts have been brought forth in defense of this theory and material from the field of physiologic chemistry, physiology, pathology and bacteriology have made it clear that auto-toxemia is a problem that must receive serious consideration at the hands of the progressive physician if he keeps pace with the times. Auto-toxemia—defined—means "poisoning from a ferment or a virus generated within the body." The absorption of these harmful substances in the blood from the intestinal or genito-urinary tract often leads, like a will-o'-the-wisp, the physician away from the well-beaten track and tests to the utmost the able diagnostician.

---

*Read at the annual meeting of the North Central Ohio Medical Society held in Mansfield Ohio, March 25, 1904.

Faulty elimination is the foundation of many diseases and poisonous substances originating within the body as a result of defective elimination, or of a putrefactive or fermentative process or perhaps both, will produce symptoms that are distinct and well defined.

Auto-toxemia is one of the accidents that arise from disturbances of nutrition, and with abnormal nutrition we are sure to have disease, and the lines along which it may manifest itself are legion. It has been said by an eminent pathologist that "more diseases are produced by faulty nutrition than are produced by anatomical lesions." In fact, it is almost a rule that a disorder in nutrition comes first and the anatomical lesion follows. It is generally conceded that the natural forces of the body are able to cope with the ordinary forces within and without itself, and so long as these conditions exist there is good health. Marked illustration of auto-toxemia is found in the usual cases of cholera morbus and the so-called cholera infantum, which is rarely a cholera infantum at all, for, candidly, I think but few cases of real cholera infantum ever occur outside of the crowded districts of the large cities. The fermentative changes in milk fed to children accounts for many infections of the intestinal canal, and we should be on our guard and fortify against such conditions as early as possible and save our little patients from bilious dysentery or pseudo-cholera infantum, and really an auto-toxemia.

It is a frequent occurrence with the average physician to meet a female patient who presents a muddy complexion, cadaverous breath, cold hands and feet, who tells you of her extreme nervousness, constipation and foul-smelling menstrual waste. As a rule, in such subjects it is clearly a case of auto-toxemia. Good drainage by way of liver and kidneys, in fact general elimination of effete material stored up in her system, with a wholesome support of the body on general principles, brings about a return of health. Another case, that of the babe cutting its first teeth, suffers from auto-toxemia, though some still contend that teething brings about reflex nerve trouble, but we believe that faulty nutrition is back of the whole trouble and that the laboratory in the intestinal canal is out of harmony with the natural order of things. In recent years, a medico-legal question has come up and must remain as a reminder, for not a few deaths from convulsions as a result of auto-toxemia have been brought to our notice.

You are familiar with the appearance of the patient who reports a gradual loss of appetite, presents a tongue, large, white and dirty, mouth clammy, breath fetid and constipation habitual.

Patient is languid, feeble, and indisposed to work. Discomfort increases after each attempt to take food. Fever is preceded by chills and followed by sweating. Hepatic engorgement and jaundice are present.

You may call it what you please, but the most common scapegoat for these symptoms is malaria, though the list of names is legion. Auto-toxemia is back of it all. Diagnose by exclusion and treat accordingly, and, as a rule, both patient and physician will be made happy. These conditions are often brought about by long continued mental strains, exposure and overwork. With children and neurotic females, vertigo and convulsions often usher in and accompany auto-infections, and disappear promptly when the conditions are changed.

The fever brought on from these conditions will sometimes last a week or ten days. These are, without doubt, the typhoid fevers that are broken up by means of *special* treatments (the "Woodbridge," for instance). The physician who diagnoses and reports such a case as typhoid, and sees it recover within seven or perhaps twelve days, should, in his own mind, decide that he was mistaken, and then fix up the fences with his patron to best advantage.

There is another condition without fever, giving off many of the constitutional symptoms already outlined and directly due to non-elimination, which usually respond promptly to treatment directed to the hepatic functions. The old-time physician, who were always "rousing" up the liver, builded better than they knew when they gave their patients the large doses of calomel. They usually relieved the patient (if death did not), especially if, as an afterthought, they followed the heroic dose of calomel with a liberal dose of Epsom salts.

Havelock Ellis has, from a psychological standpoint, ably handled the subject of the irritable neurotic, which is a good side light on the subject under consideration. He pictures out two states, the conscious and the psychologic,—the first an irritable weakness, the other a deep-seated constitutional neurosis, a result of the various conditions of temporary or protracted disorders charged to auto-toxemia.

The two conditions are predisposing factors of almost distinct value. One type disturbs the balance of health so that it is easily upset and easily recovers. In the deeper type, a secondary condition to auto-toxemia has been engendered, which, like all conditions influenced by causes secondary to a great primary origin, will not yield to treatment of the primary cause alone.

Troublesome erythemas are often a condition we must contend with in intestinal infections, and they will resist all local treatment, but readily yield under the treatment directed to the gastro-intestinal irritation. The oculist is put to the test often and is at a loss to know why certain visual disturbances do not yield to his treatment. The neurologist meets his Waterloo along the same line. The rectum receives attention and all sorts of so-called special treatments are made and given. And last, but unfortunately not least, comes the gynecologist with his speculum, curette and knife. He focuses and learnedly tells us that the trouble is within the uterine walls; he curettes and generally leaves his patient a little the worse for treatment. Ovarian irritation, suppuration and the usual line of complex symptoms arise, and, like the ghost, will not down. Then comes the knife, and an unsexed woman goes forth into the world, an alien to even her home circle, lives out her time and is as hopeless as the lost spirits we read of in "Dante's Inferno."

The woman, who is listed as a neurotic and perhaps presents the usual symptoms that accompany pelvic diseases, is asked if she drinks much water. Invariably she will answer in the negative. She tells you that she rarely takes a drink of water, and, perhaps, the only liquid consumed during the day is a small cup of tea at meal time. What may we look for in such cases? Habitual constipation; fermentation in both stomach and small intestines; sallow skin; nervous, ill-tempered and pessimistic to a high degree. This woman's system needs fluid. The unquenched thirst will cause the system to drink at a fountain most convenient, and with most individuals that fountain is the intestinal tract and the fluids used are those already loaded with sewage. The individual is thus poisoned on the same principle of the person who lives and sleeps in a house where defective sanitary plumbing continually poisons the atmosphere he breathes.

An able writer has said that "The human body is both a receptacle and a laboratory of poisons." These are found in the food and are formed in the tissues and fluids of the body, and still the healthy individual may not be poisoned. They are continually being formed in the body under normal conditions, and if they accumulate in large quantities disease will result. Auto-toxemia then results from a disturbance between the formation and destruction of these internal products.

The organs of defense must necessarily be the liver, the gastro-intestinal mucous membranes, the spleen, lymph glands, adrenals, thyroids, etc. Neurologists and alienists generally tell us that the majority of those admitted into the asylums for the insane are primarily disturbed from auto-infections.

Puerperal eclampsia is the result of auto-infection, and is a fort that has been assailed by the heaviest medical artillery of the age, and still awaits the specific. The production of fermentation and putrefaction, the former mainly in the stomach, the latter in the intestines, results in many of our common ills and nervous wrecks which our congested civilization exhibits. The gastric fermentation follows the ingestion of carbo-hydrates, while putrefaction depends on the presence of albuminous bodies for its existence in the intestinal tract. The chronic tipler of mixed drinks furnishes us a good illustration of this order, for he is a walking manufacturing establishment, a laboratory for the making of toxins, and, eventually, like "Jones of Binghampton," he "pays the freight." His symptoms are of diminished physical resistance, dyspepsia, perhaps obesity, inability to stand continued effort, neuralgia, rheumatism, and all manner of pains and muscular weakness. He becomes a hypochondriac, is generally impotent and has rectal and prostatic trouble. You have all seen him and see him every day, and with the interpretation of the handwriting on the wall, he, like Belshazzar, may learn what the end will be. Miracles will not save him, and his only hope for existence in this world is help at the hands of the intelligent physician.

Diagnosis in conditions of auto-toxemia is not so simple as it may first seem. In hereditarily defective or acquired neuropathic constitutions we must be on guard, and, again, the auto-toxemias of puberty often temporarily initiate seemingly serious neuroses and psychoses. The same is true of the climacteric end of the senile period. Grave auto-toxemias, resembling tabes and allied conditions, are often misleading.

The question of therapeutics in auto-toxemia will depend on the type, surroundings, diet and plenty of water, we care not how nor when. Aerated waters of the lithia class are beneficial; good spring water is perhaps as good as any, but water these patients *must* have, and plenty of it. The anti-toxic effect of creosote by destroying myriads of intestinal toxins, which swarm there, proves its virtues, and, we believe, in this direction lies the sole effect of creosote in our tuberculosis patients. The line of intestinal antiseptics is so extensive that we scarcely have a limit, but, in our hands, we rate, in regular order, the following:

Water, calomel, resorcin, salol (and the salicylates generally), betanaphthol, benzosol, etc. With such remedies, alone or in combinations, as the judgment of the intelligent physician decrees, much good can be done in the above outlined cases.

